# Securing Iris Recognition with Fully Homomorphic Encryption: Dimensionality Reduction in Iris Codes

**DOI:** 10.3390/s26165254

**Published:** 2026-08-19

**Authors:** Surendra Singh, Priyanka Das, Mahesh K. Banavar, Faraz Hussain, Vishnu Naresh Boddeti, Stephanie Schuckers

**Affiliations:** 1 Department of Electrical and Computer Engineering, Clarkson University, Potsdam, NY 13699, USA; prdas@clarkson.edu (P.D.); mbanavar@clarkson.edu (M.K.B.); fhussain@clarkson.edu (F.H.); sschucke@clarkson.edu (S.S.); 2 Department of Computer Science and Engineering, Michigan State University, East Lansing, MI 48824, USA; vishnu@msu.edu

**Keywords:** iris recognition, IrisCode, dimensionality reduction, privacy-preserving

## Abstract

Biometric recognition systems are fundamental to modern identity management and access control. However, the security and privacy of these systems are severely compromised when adversaries gain access to stored biometric templates, given the immutable link between individuals and their biometric traits. This study presents a benchmark analysis of the trade-offs between dimensionality and system performance in the context of securing iris biometric templates using Fully Homomorphic Encryption (FHE). We examine the impact of Principal Component Analysis (PCA) as a dimensionality reduction technique to enable encrypted-domain computation while maintaining recognition accuracy and reducing computational overhead. In lieu of introducing a new dimensionality reduction technique, this study rigorously benchmarks the applicability of PCA in balancing computational efficiency and recognition accuracy for biometric systems operating under Fully Homomorphic Encryption (FHE) constraints. We evaluate our method on various iris databases, including CASIA-V1, CASIA-V3, UBATH, and IITD. Our approach achieves a 100% True Acceptance Rate (TAR) on CASIA-V1, CASIA-V3, and UBATH and a 99.38% TAR on the IITD database at a 0.1% False Accept Rate (FAR), with a feature dimensionality of 250. This work advances iris recognition security by combining privacy measures with dimensionality reduction for improved authentication accuracy.

## 1. Introduction

To prevent identity theft and achieve higher security in a variety of applications, such as securing IT systems and crossing international borders, a trustworthy identity management system is required. The integration of biometric recognition adds an additional layer of security, ensuring that sensitive information remains protected against unauthorized access.

To ensure integrity and support widespread adoption, several security-related challenges in biometric recognition systems still need to be addressed [1], such as the following: (i) Privacy Concerns: the use of biometric technology raises privacy concerns, as biometric templates contain sensitive personal information that could be exploited by hackers; (ii) Data Breaches:in the event of a data breach, the entire biometric database could be at risk of compromise; (iii) Misuse of Biometric Data: there is a risk that biometric data may be misused for purposes such as targeted attacks or other illegal activities.

Iris recognition is valued in the biometric field for its universal presence and distinctive entropy-driven patterns, providing an effective and stable identifier. It offers advantages such as its robustness as a substitute for conventional visual recognition methodologies, like face recognition. Furthermore, iris recognition demonstrates efficacy in expediting the search process within extensive databases [2].

For these measures, it is crucial to protect the iris database without compromising the accuracy of the system [3]. In this study, we present an efficient iris recognition system with enhanced security through the integration of Fully Homomorphic Encryption (FHE). We present a benchmark analysis focused on dimensionality reduction for efficient and secure iris recognition under FHE. FHE enables the computation of biometric data directly on encrypted biometric data, significantly improving privacy and security. However, the high dimensionality of raw iris templates poses a major computational challenge, rendering FHE impractical without prior dimensionality reduction. To address this, we employ Principal Component Analysis (PCA) to compress the feature space while retaining essential discriminatory information. We systematically evaluate iris recognition accuracy across multiple feature dimensions, highlighting the trade-offs among template size, computational feasibility, and recognition performance. This benchmark provides practical insight into selecting appropriate feature dimensions to support real-world deployment of FHE in biometric systems.

Template protection techniques can be categorized into two main groups: feature transformation (including methods such as salting and non-invertible transformation [4]) and biometric cryptosystems (which involve key binding [5] and key generation). However, these methods can cause degradation of iris recognition accuracy due to inherent variability in biometric data [6]. Homomorphic encryption is a promising solution to protect biometric data [7]. It offers several advantages over existing techniques, including strong security and privacy guarantees. Homomorphic encryption allows users to perform specific algebraic operations on ciphertext (encrypted form of a plaintext message) directly and decrypt the result, similar to applying the same method directly to plaintext [8,9,10,11]. Homomorphic encryption offers many advantages in privacy, security, and flexibility for biometric templates. Firstly, it enables the templates to be securely stored while allowing computations to be performed on them. This means that the privacy of the data can be maintained even during biometric matching. Secondly, the encryption of the templates minimizes the possibility of unauthorized access, thus enhancing the security of the system [12]. This research presents a benchmark study evaluating the feasibility and performance trade-offs of an FHE-based, privacy-preserving iris authentication system that addresses both template security and computational constraints. The key components assessed in this system are as follows:

1. Dimensionality Reduction:PCA is employed to reduce the size of iris templates, making encrypted-domain computation viable by lowering the computational burden associated with high-dimensional biometric data. While PCA is a well-established technique, in this work it is systematically analyzed under FHE constraints to study the trade-offs among feature dimensionality, recognition performance, and computational feasibility.
2. Efficient Encrypted Computation: A batching technique, as proposed by Boddeti [13], is incorporated to enable simultaneous multiplication of multiple ciphertexts, significantly reducing the overall processing time. This allows practical encrypted-domain operations despite the inherent overhead of FHE.
3. Encrypted-Domain Matching: The system computes the inner product between encrypted biometric reference and query templates entirely within the encrypted domain, preserving data confidentiality throughout the matching process. The use of inner-product-based similarity is motivated by its compatibility with homomorphic operations, enabling efficient computation without requiring complex non-linear functions.
4. Comprehensive Evaluation: Our method is evaluated on four publicly available iris databases: CASIA-V1, CASIA-V3, UBATH, and IITD. System performance is assessed using standard biometric metrics, including the True Acceptance Rate (TAR) and Equal Error Rate (EER), to benchmark the trade-offs among dimensionality, accuracy, and computational efficiency. In addition, we perform cross-database evaluation (training on CASIA-V3 and testing on other datasets) to analyze generalization performance.
5. System-Level Contribution: The primary contribution of this work lies not in the development of a novel dimensionality reduction algorithm but in a systematic benchmark study of iris recognition within the constraints of FHE. In contrast to prior approaches that rely on heuristic block-wise reduction methods, the proposed framework employs PCA as a statistically grounded technique to retain discriminative information while substantially reducing the dimensionality of the template. Furthermore, the integration of PCA with SEAL-based encrypted-domain matching establishes a practical and reproducible pipeline for privacy-preserving biometric authentication. This study therefore provides a comprehensive evaluation of the interplay among feature dimensionality, recognition performance, and computational overhead in the encrypted domain.

## 2. Related Work on Homomorphic Encryption for Biometrics

Bassit et al. [14,15] proposed multiplication-free methods for matching biometric templates. This helped reduce the computational complexity of prior work that needed homomorphic encryption. Sperling et al. [16] proposed a method to fuse multiple biometric templates directly in the encrypted domain through a linear projection followed by normalization. Engelsma et al. [17] proposed to reduce the computational complexity of encrypted template-matching through a synergistic combination of dimensionality reduction of biometric templates and an encoding scheme that is better suited for scalable matching. This scheme was demonstrated on face and fingerprint templates. The dimensionality reduction technique used in this research was DeepMDS++. Sumalatha et al. [18] integrated CNN-based fingerprint recognition with CKKS-based FHE using TenSEAL [19], enabling authentication in the encrypted domain. Furthermore, recent research has extended FHE-based template protection to other biometric modalities, including fingerprints, gait recognition [20], and face recognition [21]. These studies highlight the growing interest in applying homomorphic encryption for secure biometric authentication while maintaining recognition performance and privacy guarantees. In this paper, we explore the dimensionality of the enrollment and verification templates in iris recognition using PCA and study its accuracy as a function of the feature dimension.

Panda [22] proposed the homomorphic PCA algorithm (HPCA), which demonstrates a novel approach to performing PCA on encrypted data using the CKKS homomorphic encryption scheme. Unlike traditional methods, the server in the client–server model avoids computationally intensive matrix operations such as multiplication and inversion on encrypted data, thereby reducing the overhead. Experimental results in multiple datasets highlight the effectiveness of HPCA, with R^2^ scores used to evaluate the accuracy of reconstruction, demonstrating its potential for privacy-preserving machine learning. Although their work did not specifically target biometrics, the methodology illustrates how encrypted-domain feature transformation can be applied to secure sensitive data. In contrast, our proposed iris recognition framework applies PCA on plaintext features to reduce dimensionality while minimizing data loss, followed by FHE to protect the resulting iris templates.

In the context of biometric recognition, homomorphic encryption serves as a bio-cryptosystem by directly applying encryption techniques to biometric data, distinguishing itself from non-invertible transformations [23]. It meets the requirements outlined in ISO/IEC 24745 [24], which offers guidance for the protection of biometric information through various criteria for confidentiality, integrity, and renewability/revocability during storage and transfer [25]. Biometric applications that integrate homomorphic encryption algorithms stand to benefit from enhanced data security, and the prospect of standardization is expected to further amplify their integration and acceptance within the field [26]. Several papers have looked at face recognition systems with FHE  [27,28,29] to safeguard users’ privacy. Boddeti, proposed face matching over encrypted face templates using FHE, introducing a privacy authentication scheme to match the binary face templates 128-bit and 512-bit in the encrypted domain. The process is divided into enrollment and authentication. During the enrollment phase, the user generates a public and private key to encrypt the feature vector of the face. The public key, encrypted feature vector, and user identity label are transmitted for matching. However, the private key remains on the client device during the authentication phase. In our work, this method is adopted for iris recognition.

In related work on fusion FHE systems that combine with the iris, Bayer et al. [30] employed deep feature representations and derived 512-dimensional vectors for both modalities, which facilitated efficient fusion and distance calculation in the encrypted domain and improved privacy-preserving biometric authentication. Vallabhadas et al. [31], proposed an authentication mechanism that is more precise, requires less computing time, and protects template privacy, which is a client–server model that uses a combined feature vector of iris and fingerprint. Since the original feature vectors were very large (10,240 for iris and 4096 for fingerprint), they applied block-wise compression with an optimal block size of 4, reducing the fused vector to 3584 dimensions while balancing security, noise, and efficiency. Morampudi et al. [32,33] developed bimodal privacy-preserving authentication using fingerprint and iris, and these templates were concatenated to form the fused reference template. They also employed block size reduction to compress the fused binary template, demonstrating the conversion of a vector of size 1 × 16,072 into a vector of size 1×4018. Singh et al. [34] fused the feature vectors of iris and face, encrypted the fused feature vector, and performed matching. The final iris code matrix, with dimensions 128×512, was transformed into a vector of size 65,536 × 1 using a row-wise flattening operation. PCA was then applied to reduce the iris feature vector to 250 dimensions. The face feature vector consisted of 512 features, resulting in a final fused feature vector of length 1012.

Morampudi et al. proposed a technique that can encrypt and calculate the Hamming distance of iris characteristics in 2560 dimensions within approximately 0.0185 s. This method achieved an Equal Error Rate of 0.19% for the CASIA-V 1.0 database. Block size reduction is used to reduce the size of the iris template. For example, if the total number of bits in the iris templates is 10,240, the template is reduced by a block size of d=10. The total number of bits is divided by *d*, resulting in a final template size of 1024.

Morampudi et al. proposed a system called SvaS (secure and verifiable classification-based iris authentication system) FHE that aims to achieve privacy-preserving (PP) training and PP classification of nearest neighbor and multi-class perceptron models for iris authentication. The system involves four entities: the client device, cloud server, authentication server, and public verifier. Block size *d* reduction iris template compression is performed, and matching is performed using NN (nearest neighbor) and MCP (multi-class perceptron) on the encrypted data. PNN (private nearest neighbor) and PMCP (private multi-class perception) provide privacy not only to the iris templates but also to the model by performing both PP training and PP classification. The model is only accessible to the server, and the training and test instances are only accessible to the client device, providing an added layer of security. This method achieved 0.87% EER on CASIA-V1, 0.95% EER on CASIA-V3, 1.02% EER on IITD, and 0.92% EER on the SDUMLA-HMT database, each with a template dimension of 2560.

This paper provides a comprehensive evaluation of PCA for reducing dimensions in iris biometric template compression, particularly within privacy-protecting frameworks using FHE. Unlike earlier methods that rely on basic reduction strategies such as pixel- or bit-skipping, which are not statistically robust and often compromise important discriminative information, PCA offers a statistically sound method that retains the most critical variance in the feature space. By projecting high-dimensional iris templates into a lower-dimensional subspace, PCA significantly reduces template size while preserving recognition accuracy. This evaluation assesses PCA across various public iris datasets and different dimensional settings, demonstrating its ability to meet the dual goals of computational efficiency and reliable biometric matching. The findings highlight PCA suitability as a core element for secure biometric recognition systems in encrypted domains. This improvement not only optimizes template size but also boosts the robustness and efficiency of iris recognition systems.

## 3. Fully Homomorphic Encryption for Iris Authentication with Principal Component Analysis PCA

Based on previous research on iris recognition using FHE [35], we encrypt iris templates using FHE and conduct matching directly in the encrypted domain. Table 1 illustrates a comparison between the proposed method and previous related research. In the paper, we study the impact of reducing the dimensionality of iris templates, which involves using PCA. PCA outperforms block size reduction methods for iris template size reduction because it can effectively compress the relevant information into a smaller set of principal components, while still retaining the distinctive features of the iris template. In our study, we applied PCA to train on the CASIA-V3 database using iris code dimensions of 128×512. Subsequently, we evaluated the performance of this trained PCA by assessing its variance across other databases—specifically, the CASIA-V1, IITD, and UBATH databases—in a cross-database testing scenario.

**Figure 1:**
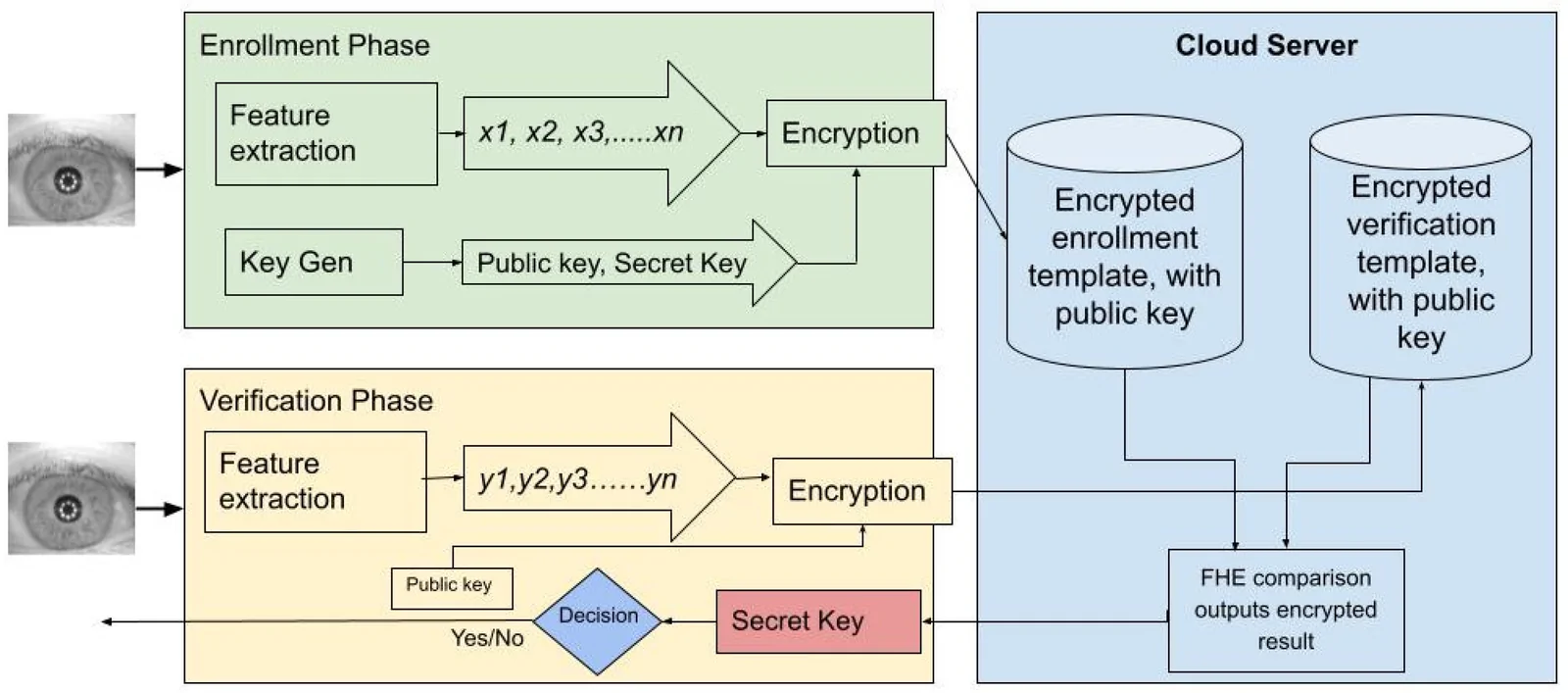
Enrollment and verification processes for iris matching with FHE. During enrollment/verification, OSIRIS [36] processes the feature extraction from the iris image. Keygen generates the public and secret keys, and the encrypted template and the public key are sent to the cloud server. The server performs the matching process, and the encrypted matching result is then sent back to the verification device. The secret key is used to decrypt the matching score.

Figure 1 illustrates the recognition process using FHE, with two stages: enrollment and verification. During the enrollment process, public and secret keys are generated and the enrollment template is encrypted. The encrypted enrollment template and public key are sent to the server. Next, during verification, an encrypted verification template is generated to the client and passed to the server. The matching score is computed by comparing the enrollment and verification encrypted templates using the public key. Lastly, the encrypted matching score is returned to the client device and decrypted using the secret key.

Moreover, the architecture is suited for practical deployment, where the matching process is offloaded to a cloud server. This approach minimizes the computational load on client devices, making it appropriate for lightweight or resource-constrained devices. Since the biometric templates are transferred from the client device to the cloud server for matching, the small size of the templates ensures that data transfer remains efficient, even in environments with limited network bandwidth. This combination of compact templates and cloud-based matching makes our system not only secure but also highly scalable, providing a robust solution for real-world biometric authentication systems.

### 3.1. Approach

In this study, we adhered to the procedures and algorithm given by Boddeti, which was designed for face recognition. We have modified it for iris recognition. This research paper includes a description of the algorithm as well as its implementation with iris data, performance evaluation, algorithm fine-tuning, and future enhancement opportunities.

### 3.2. Data Preparation

The proposed system was evaluated using four publicly available iris datasets:

- CASIA-V1  [37]: Contains 756 iris images from 108 subjects, with seven images captured for each eye.
- CASIA-V3  [38]: A challenging dataset characterized by eyelid and eyelash occlusions, variations in iris rotation, and limited visible iris area. It reflects realistic acquisition conditions where noise is common. The dataset includes 395 subjects, comprising 1307 right-eye and 1332 left-eye images.
- IITD  [39]: Consists of multiple iris images collected from 224 subjects.
- UBATH: Contains 1000 iris images from 50 subjects.

**Figure 2:**
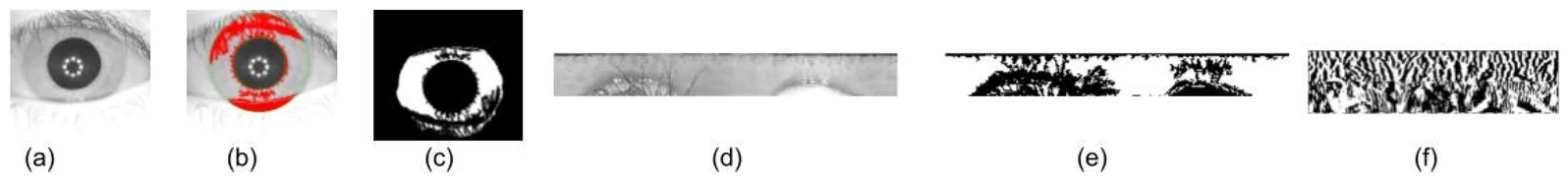
OSIRIS feature extraction: (**a**) original iris image, (**b**) segmented image, (**c**) associated binary mask, (**d**) normalized image, (**e**) normalized mask, and (**f**) iris code.

Feature extraction was performed using the OSIRIS toolkit, which follows a four-stage pipeline: segmentation, normalization, mask generation, and encoding. Figure 2 illustrates this process. Multiple iris-code and mask dimensions were generated for analysis, with the corresponding sizes summarized in Table 2.

To ensure an unbiased evaluation and prevent information leakage, a subject-disjoint protocol was adopted. Specifically, 50% of the subjects from the CASIA-V3 dataset were used to train the PCA transformation, while the remaining subjects were reserved exclusively for testing. This guaranteed that no identity would appear in both the training and evaluation sets, enabling a fair assessment of generalization performance.

### 3.3. Homomorphic Encryption Keys and Encrypted Template Details

The Microsoft SEAL library [40] is a tool that enables the implementation of FHE. This encryption method has several components that play specific roles in the encryption process. To give an idea of the size of the encryption data, Table 3 shows the dimensions of the key and encrypted template. We lack a comparison of the template and key sizes with prior work, because researchers did not provide information on key sizes and the encrypted iris template.

- Public Key: The public key is used to encrypt the data that needs to be encrypted and computed. The encrypted data is then sent to a third party, such as a cloud server, that performs computation on the encrypted data with a public key.
- Secret Key: The secret key is typically stored on the client device and is used to decrypt the result of the computation after it is sent back to the client device.
- Galois Key: The Galois key is used to perform the Galois operation on ciphertext. This key is sent to a third-party server along with the public key. In this implementation, the Galois key is used to perform shifting elements in a vector or reordering the bits in an encrypted integer.
- Relin Keys: The relinearization key is used to reduce the degree of a ciphertext polynomial after performing a homomorphic multiplication operation. This key is sent to a third-party server along with the public key.

### 3.4. Final Iris Code Creation and Matching Process

As outlined by John Daugman, the Hamming distance (HD) evaluates the dissimilarity between binary iris codes and their corresponding masks. The incorporation of a mask in Equation (1) enables selective comparison by filtering out noisy or occluded regions, thereby emphasizing reliable iris features. To follow standardized iris recognition procedures, we utilize iris codes and masks generated using OSIRIS, where the iris code dimensions are 128×512 and the mask dimensions are 64×512.

(1) $$HD=\frac{\left||(iriscode1⊗iriscode2)⋂mask1⋂mask2|\right|}{\left||mask1⋂mask2|\right|}$$

**Figure 3:**
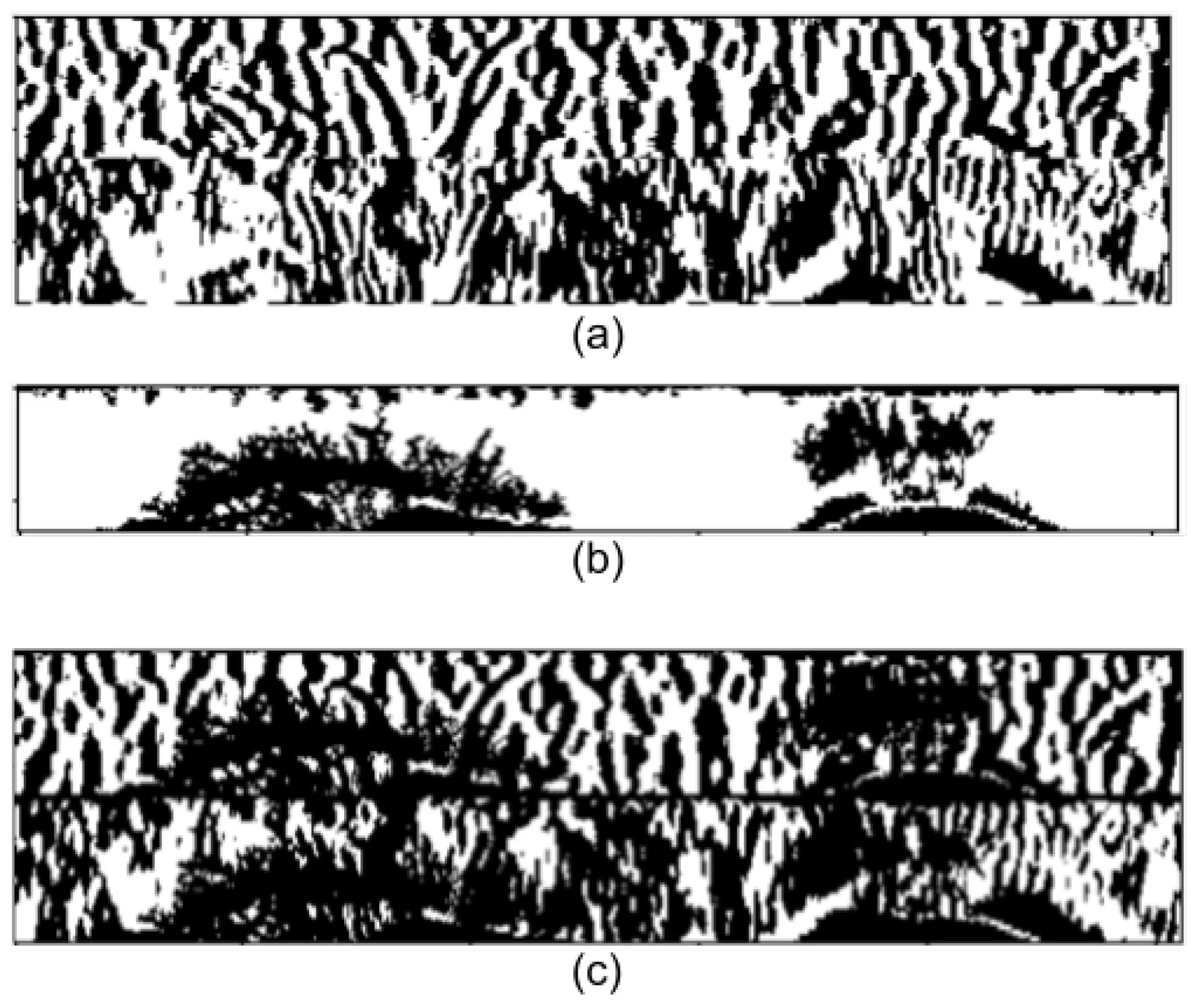
Example of (**a**) iris code, (**b**) its associated normalized mask, and (**c**) logical AND operator between iris code and normalized mask.

In this work, the first two filters of the iris code (128×512) are used. Since the normalized mask dimension is 64×512, the mask is concatenated to match the iris code dimension of 128×512. To generate the final iris representation, a logical AND operation is applied between the iris code and the corresponding mask, as illustrated in Figure 3. This operation ensures that only valid iris regions contribute to subsequent processing.

(2) enrollment=iriscode1⋂mask1

(3) verification=iriscode2⋂mask2

To generate the enrollment and verification templates, Equations (2) and (3) apply the logical AND operator to merge the iris code and mask. In these equations, ‘iriscode1’ and ’mask1’ denote the enrollment iris code and mask, while ‘iriscode2’ and ‘mask2’ represent the verification iris code and mask, respectively. This masking step reduces the influence of variations such as illumination changes, occlusions, and noise, thereby improving robustness. Following template generation, both gallery and probe templates are encrypted before being transmitted to the cloud server for matching. Importantly, only encrypted templates are stored in the database, ensuring data confidentiality.

To further improve computational efficiency, PCA is applied to reduce the dimensionality of both enrollment and verification templates prior to encryption. Equation (4) describes the transformation and dimensionality reduction process used to generate compact iris templates suitable for FHE.

**Figure 4:**
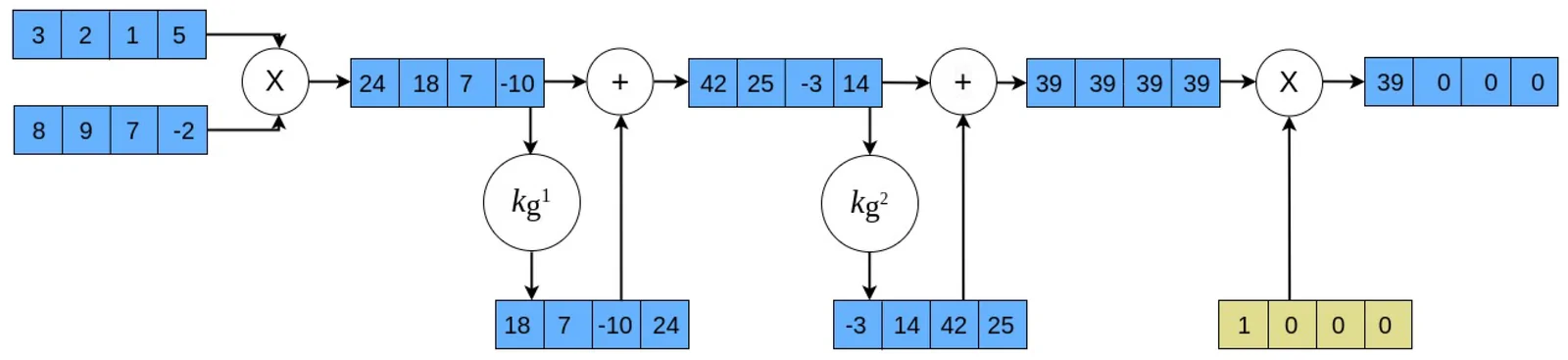
Using batch processing and homomorphic computing, multiple items are encrypted into a single encrypted block to calculate the inner product of vectors. The encrypted block’s individual elements cannot be accessed individually during the batching process. As a result, following the Hadamard product, the total of the elements contained within the encrypted block may be determined by repeatedly performing cyclic rotations and additions.

HD (Equation (1)) is commonly used for iris recognition; its direct implementation in the encrypted domain is computationally inefficient due to the requirement of bitwise XOR operations, masking, and division operations, which are expensive under FHE constraints. Therefore, instead of performing masked HD in the encrypted domain, we adopt a homomorphic inner-product-based similarity measure, as illustrated in Figure 4. This approach is more compatible with homomorphic encryption operations, as it primarily relies on additions and multiplications, which are efficiently supported in FHE schemes.

This formulation enables efficient similarity computation between encrypted enrollment and verification templates while leveraging both iris code and mask information. Consequently, the proposed framework supports secure iris recognition in the encrypted domain, while maintaining robustness and accuracy under varying environmental conditions and image quality variations.

(4) $$\begin{array}{cc} IrisCode & =128\times 512\\ IrisMask & =(64\times 512)+(64\times 512)\\ IrisMask & =128\times 512\\ FinalIrisCode & =IrisCode⋂IrisMask\\ ReducedIrisCode & =PCA(FinalIrisCode)\\ normalization & =l2norm(ReducedIrisCode) \end{array}$$

### 3.5. Reducing Iris Template Size

We experimented on two different dimensions of the iris code. Table 2 explains the distinct dimensions of the mask and iris code, along with the final vector size. To perform matching between two templates, there may be rotational inconsistencies. To address this issue, one template was shifted left and right bit-wise. We conducted ±7 shifts on the gallery dataset to match the probe against all shifted samples.

The size of the iris template affects how efficiently the matching algorithm performs. The size of the iris template can be decreased, m thereby decreasing the storage and computational complexity of the overall system. We performed PCA to reduce the dimensionality of the iris template. The main goal of applying PCA was to identify the most crucial details in the data [41]. We performed a vectorization process on the final iris code matrix of dimensions 128×512 by employing a row-wise flattening operation, thereby transforming it into a vector of size 65,536 × 1, which we used for training for PCA. We randomly selected 50% of the CASIA-V3 gallery data for training, while the remaining data were used for testing. We saved the trained PCA matrix for transforming the enrollment and verification templates. In this study, we reduced the 65,536-bit binary vector to 50-, 100-, 150-, 200-, and 250-dimensional vectors and performed accuracy analysis on each. To further assess generalization capability, the trained PCA derived from CASIA-V3 was also applied to cross-database evaluation using the CASIA-V1, IITD, and UBATH datasets. This cross-dataset evaluation enabled analysis of the robustness of the reduced feature space across different acquisition conditions and sensor variations.

### 3.6. Enrollment and Verification

The iris recognition system consists of two phases: enrollment and identification/verification. During enrollment, the iris template is obtained and encrypted before being transferred to the cloud server with a public key. In the authentication phase, the iris template is obtained from the probe iris image, encrypted, and then sent to the cloud server with a public key. To perform the matching between the two encrypted templates, we matched the probe against all shifted gallery samples and returned the matching result to the client device. Algorithms 1–3 explain the steps involved in the enrollment, verification, and matching.
**Algorithm 1** Enrollment processGenerate public, galois, relin, and secret keys.The client device generates the enrollment iris template from the reference iris image using the OSIRIS toolkit, extracting the iris features (iris code and mask).Apply pre-trained PCA and reduce the template dimension.Perform the l2normalization.The client device transmits all 15 encrypted enrollment templates (to resolve rotation inconsistencies) to the cloud server along with a class label and public, Galois, and relin keys. The secret key remains in the client device.

**Algorithm 2** Verification process
The client device generates the verification iris template using the OSIRIS toolkit, extracting the iris features (iris code and mask).Apply pre-trained PCA and reduce the template dimension.Perform the l2normalization.The client device transmits the verification encrypted template to the cloud server along with a class label.


**Algorithm 3** Matching process
Cloud servers perform matching between enrollment and verification encrypted templates.Homomorphic inner product used to perform matching between gallery and probe templates.Matching is performed using a homomorphic inner product between the encrypted template of the probe and all 15 rotated encrypted templates in the gallery.After performing the matching algorithm, the client server returns all 15 encrypted matching scores to the client device.The client device decrypts all 15 matching scores by using the secretkey and makes the decision based on the highest score.


### 3.7. Secure Matching

While masked HD is the standard similarity measure for iris recognition, its direct implementation in the encrypted domain is computationally inefficient due to the reliance on bitwise XOR, masking, and division operations. To address this limitation, the proposed framework adopts an inner-product-based similarity measure that is naturally compatible with homomorphic encryption primitives.

Masking is applied in the plaintext domain prior to encryption, after which PCA and l2 normalization are performed. Under this representation, the inner product between feature vectors approximates cosine similarity, preserving the relative ordering of genuine and impostor scores. This provides a practical and efficient alternative to masked HD in privacy-preserving settings.

In this work, we consider a one-to-one verification scenario. We implement the homomorphic inner-product matching algorithm proposed by Boddeti. Figure 4 provides a visual overview of the matching pipeline, and Algorithm 4 presents a step-by-step description of the procedure. The algorithm operates on two encrypted vectors: a gallery vector *x* and a probe vector *y*. These vectors are first multiplied element-wise, and the resulting encrypted vector is then subjected to a sequence of cyclic rotations and additions, performed *l* = $\log(w^{n})$ times, where denotes the length of the encrypted vectors. This iterative rotation-and-accumulation process efficiently computes the encrypted sum of products, yielding the homomorphic inner product.
**Algorithm 4** Homomorphic inner product  1:**procedure** ENCRYPTED INNER PRODUCT(x,y)  2:      ε(x)←f(x,θe)                       ▹ Gallery Encoding  3:      ε(y)←f(y,θe)                          ▹ Probe Encoding  4:      ct←Multiply(ε(x),ε(y))                       ▹ Multiply  5:      **for** i={0,…,l} **do**  6:            $ct\leftarrow ct+K_{g^{i}}\left(ct\right)$                      ▹ Cyclic Rotation  7:      **end for**  8:      **return** ct                   ▹ The element in the first slot is the desired inner product.  9:**end procedure**

## 4. Experiments and Results

Experiments were conducted on the CASIA-V1, CASIA-V3, IITD, and UBATH iris datasets. For each dataset, one image from a subject was treated as the probe, while all remaining images served as gallery samples. This setup ensured a consistent one-to-one verification protocol across all datasets.

Performance evaluation was based on decision thresholds computed independently for each dataset and operating point. Thresholds were estimated using score distributions derived from the subject-disjoint evaluation protocol, with particular attention to avoiding optimistic bias. Standard biometric operating points were considered, including 0.1% FAR and 0.01% FAR, enabling consistent comparison across datasets and dimensionality settings.

For each dataset and feature dimension, the selected threshold was applied uniformly to all genuine and impostor comparisons. This ensured that the reported TAR and FAR values reflected the behavior of the system under fixed, dataset-specific operating conditions rather than thresholds tuned per comparison or per subject. The resulting performance metrics therefore provide an unbiased and reproducible assessment of system accuracy.

### 4.1. Accuracy Analysis

Although the system follows a one-to-one verification protocol, multiple circular shifts (15 rotations) are applied to compensate for iris misalignment. The final matching score is taken as the maximum similarity across all rotated comparisons, which is consistent with standard iris recognition practice.

**Figure 5:**
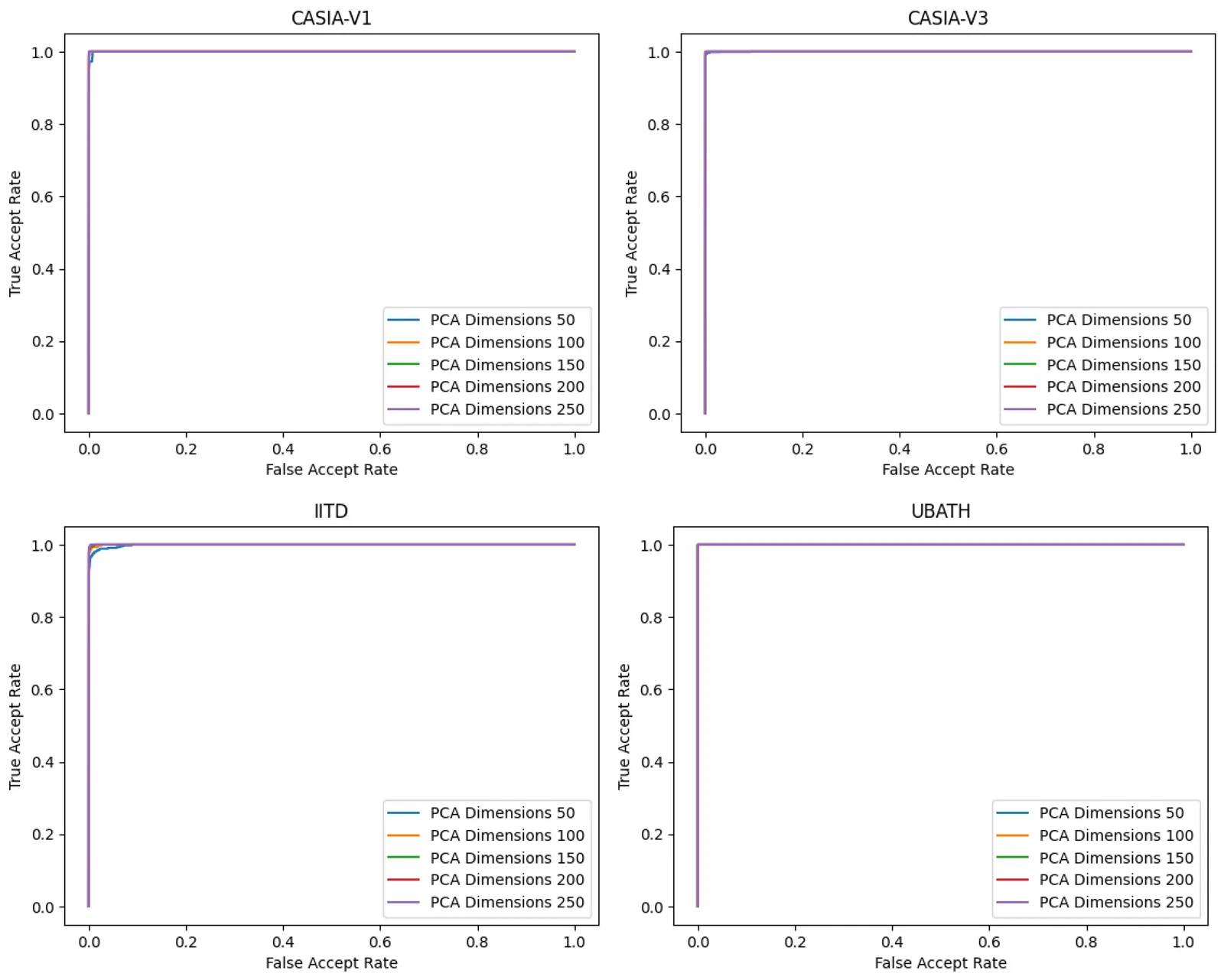
ROC curves illustrating recognition performance for the original 65,536-bit iris code (128×512) and PCA-reduced feature representations across the CASIA-V1, CASIA-V3, IITD, and UBATH databases. The curves demonstrate the trade-off between dimensionality reduction and recognition accuracy. Performance is evaluated using TAR at different FAR operating points.

The performance trends observed across datasets are summarized in Table 4 and Figure 5. For the IITD dataset, the TAR increases as the feature dimensionality grows. At a feature length of 250, the system achieves a TAR of 98.38% at 0.1% FAR, demonstrating the effectiveness of the proposed approach. The CASIA-V3 dataset reaches a TAR of 100% at 0.1% FAR with a feature length of 250. Similarly, CASIA-V1 attains a TAR of 100% at 0.1% FAR once the feature length reaches 150 or higher. The UBATH dataset also achieves a TAR of 100% at 0.1% FAR with as few as 50 features, which may be attributed to the relatively lower noise level in UBATH compared to the other datasets.

### 4.2. Accuracy Analysis for Different Iris Code Dimensions

**Figure 6:**
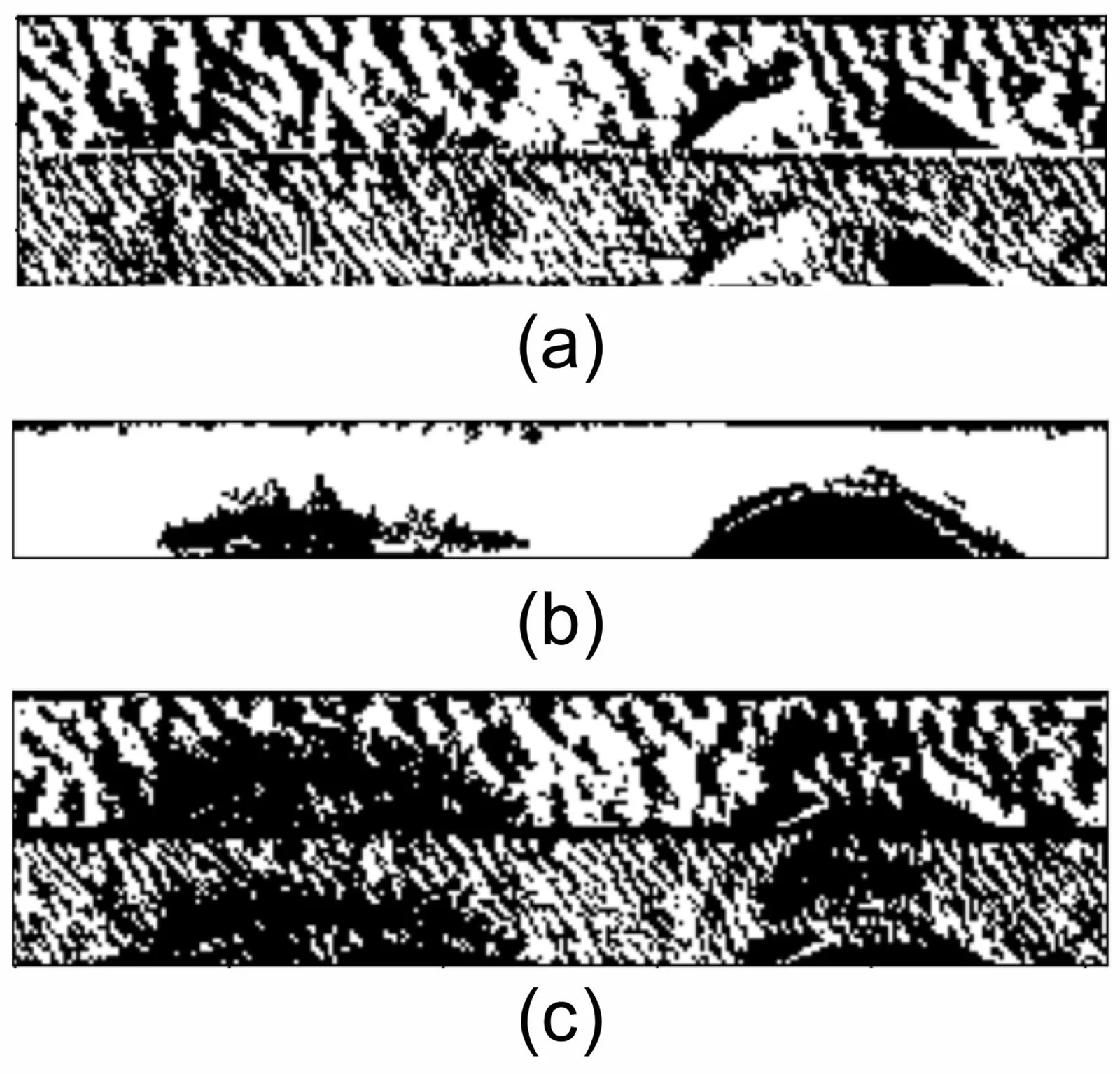
Example of reduced-dimension iris code and mask generated by OSIRIS where (**a**) iris code, (**b**) its associated normalized mask and (**c**) logical AND operator between iris code and normalized mask. Final iris created with AND operation of 32×256 mask and 64×256.

In this section, we generated two different iris code dimensions of the CASIA-V3 iris database, where Figure 6 illustrates the final iris code. Table 2 shows the different dimensions of iris feature extraction from OSIRIS. The default OSIRIS configuration is set to generate a 64×512-pixel normalized image, and two Gabor filters are used to generate a 128×512-pixel iris code. In OSIRIS, the default Gabor filter kernel is configured as a 9×15 matrix. Since our normalized iris images are resized to 32×256, the Gabor filter kernel is proportionally reduced to 6×7 to align with the new spatial resolution. This proportional adjustment preserves the spatial-frequency response of the filter relative to the image scale, ensuring that both the original and resized iris images encode comparable texture information for fair and consistent iris code generation. After modifying the filter size, the normalized iris image remains 32×256, and the resulting iris code generated through phase quantization is 64×256. Table 4 and Table 5 provide information on the accuracy of iris recognition at different iris dimensions of the CASIA-V3 database. For iris code dimensions of 64×256, the study TAR was noted at 0.1% FAR with 100 or more feature components. Similarly, for iris dimensions of 128×512, 100% TAR at 0.1% FAR was noted at 250 feature components. Table 5 explains the performance (TAR at 0.01% and 0.1% FAR) of various feature dimensions. A comparison of the results for both iris code dimensions reveals that 64×256 exhibits higher accuracy compared to 128×512. We assumed that during the generation of the 64×256 iris code from OSIRIS, modifying the Gabor filter did not eliminate essential features. Given that the final feature vector size was 16,384, which is four times smaller than the iris code size of 1285×512 (65,536 vector size), it suggests that the iris code dimensions of 64×256 lead to higher accuracy at 200 and more feature dimensions.

### 4.3. Comparative Analysis with Previous Research

In comparison to our study, Morampudi et al. utilized the University of Salzburg Iris Toolkit to extract iris codes with dimensions of 20×512. They implemented a block size reduction method to decrease the iris template size from 10,240 to 2560. Morampudi et al. [3] applied Hamming distance as the matching algorithm. The achieved EERs across different databases were as follows: CASIA-V1 - 0.19%, CASIA-V3 - 0.39%, IITD - 0.99%, and SDUMLA-HMT - 0.28%.

Morampudi et al. applied NN (nearest neighbor) and MCP (multi-class perceptron) matching algorithms. They achieved minimum EER with the NN matching algorithm across different databases, as follows: CASIA-V1 - 0.87%, CASIA-V3 - 0.95%, IITD - 1.02%, and SDUMLA-HMT - 0.92%.

Table 1 illustrates the comparison of template sizes using our proposed PCA compression method and previous research template size reduction techniques. Given the various differences in the implementation of iris recognition algorithms in prior work and the unavailability of the code, we implemented the block size reduction method in order to compare our approach with the block size method. To implement the block size reduction proposed by previous researchers, we divided the final bit count (65,536) by 250 and 1200, yielding template vector sizes of 263 and 54, respectively. In this study, after applying PCA, we selected a template size in the range of ‘50–250’. To implement the block size reduction method, we selected 54 as the minimum and 263 as the maximum template size, as well as comparing it with our proposed PCA compression method, where we used 50 as the minimum and 250 as the maximum dimensions. With block size reduction at a dimension of 263, we achieved EER across different databases as follows: CASIA-V1 - 0.052%, CASIA-V3 - 0.208%, IITD - 2.246%, and UBATH - 1.676%. In comparison with PCA at the dimension of 250, we achieved improved EER over block size reduction for different databases as follows: CASIA-V1 - 0.011%, CASIA-V3 - 0.039%, IITD - 0.260%, and UBATH - 0.00%. Additionally, in our approach, the template dimensions were much smaller compared to previous research, and we still achieved superior performance.

### 4.4. Computational Efficiency

To evaluate the computational efficiency of the proposed system, we performed a one-to-one matching experiment, where one probe template was matched against a gallery template. In our setup, 15 encrypted gallery templates and one encrypted probe template were generated, requiring approximately ∼1 s for encryption. For the matching stage, the encrypted probe template was compared against all 15 encrypted gallery templates, and the corresponding matching scores were computed in approximately ∼0.5 s. The tests were conducted on a machine with an 8-core Intel(R) Core(TM) i7-10700 CPU at 2.90 GHz.

## 5. Conclusions

The objective of this study was to investigate the practical implementation of securing an iris database and performing matching on an encrypted template database using Fully Homomorphic Encryption. The analysis that we conducted showed that there is a trade-off between the size of the template and the accuracy of the system. In previous research on iris recognition using Fully Homomorphic Encryption, researchers achieved 0.39% and 0.95% EER on the CASIA-V3 database at 2560 iris code dimensions. In our method, using PCA for feature reduction on CASIA-V3, we observed that original iris dimensions of 64×256 achieved a 0% Equal Error Rate (EER) when reduced to 200 or more feature dimensions, and the iris code with original dimensions of 128×512 had an EER of 0.039% at 200 and 250 feature dimensions. We also experimentally trained PCA on the CASIA-V3 database and tested it with other databases: IITD, CASIA-V1, and UBATH. We achieved significantly low Equal Error Rates (EERs) of 0.26%, 0.01%, and 0.00% for the respective databases. We also achieved a 100% TAR on CASIA-V1, CASIA-V3, and UBATH and a 99.38% TAR on the IITD database, at a 0.1% False Accept Rate (FAR) with a feature dimensionality of 250. Fully Homomorphic Encryption appears to be a promising solution for accurate iris matching in the encrypted domain, which may help prevent information leakage and protect user privacy.

In this study, OSIRIS was employed for feature extraction due to its standardized and widely adopted iris encoding framework. However, alternative feature extraction methods with inherently lower-dimensional representations may further reduce template size while preserving discriminative information. Future work will explore dimensionality reduction techniques such as autoencoders and deep embedding-based compression to evaluate their effectiveness in minimizing template size while maintaining recognition performance.

Additionally, further investigation is required to optimize encryption parameters, including reducing encryption key sizes and compressed template representations. Since template and key sizes directly influence the transmission time between client devices and cloud servers, future research will focus on analyzing the trade-offs among template size, recognition accuracy, and encrypted computation efficiency. Moreover, evaluating these approaches across multiple datasets and exploring large-scale deployment scenarios will help identify optimal configurations for practical privacy-preserving iris recognition systems.

## Figures and Tables

**Table 1:** Comparative analysis of the proposed methods and previous studies in terms of dimensionality reduction, template size, and recognition performance. Template sizes are reported before and after dimensionality reduction. Recognition performance is presented as EER% across multiple iris databases. Abbreviations: DR—dimensionality reduction; MA—matching algorithm; DB1—CASIA-V1; DB2—CASIA-V3; DB3—IITD; DB4—SDUMLA-HMT; DB5—UBATH; HD—Hamming distance; NN—nearest neighbor; MCP—multi-class perceptron; IP—inner product.

				Template Size		Database and Accuracy in EER (%)				
Author	DR	Tools	MA	Before	After	DB1	DB2	DB3	DB4	DB5
Morampudi et al.	Block size	USIT	HD	10,240	2560	0.19	0.39	0.99	0.28	–
Morampudi et al.	Block size	USIT	NN, MCP	10,240	2560	0.87	0.95	1.02	0.92	–
Our block size approach	Block size	OSIRIS	IP	65,537	54	0.80	0.85	2.30	–	0.32
				65,537	263	0.05	0.20	2.24	–	1.67
Our PCA approach	PCA	OSIRIS	IP	65,537	50	0.85	0.45	1.70	–	0.00
				65,537	250	0.01	0.03	0.26	–	0.00

**Table 2:** Dimensions of iris features generated using OSIRIS, showing mask size, resulting iris code dimensions after concatenation, and corresponding vector sizes used for feature representation.

Mask	Final Iris Code	Vector Size
32 by 256	64 by 256	16,384
64 by 512	128 by 512	65,536

**Table 3:** Sizes of encryption keys and encrypted templates used in the FHE framework. The table reports memory requirements for keys and encrypted enrollment and verification templates.

		Template/Key Size
Key	Public key	136.3 KB
	Secret key	68.3 KB
	Galois key	9.0 MB
	Relin key	408.5 KB
Encrypted template	Enrollment encrypted template	96.7 KB
	Verification encrypted template	96.7 KB

**Table 4:** Accuracy analysis of 65,536 (128×512) iris code dimension of CASIA-V3-trained PCA on a randomly selected 50% of gallery data and tested against CASIA-V3 (remaining 50%), CASIA-V1, IITD, and UBATH databases with different feature dimensions.

Database	PCA Dimensions	TAR at 0.1% FAR	Threshold at 0.1% FAR	TAR at 0.01% FAR	Threshold at 0.01% FAR	EER%
CASIA-V3	50	99.22	0.63	98.37	0.73	0.454
CASIA-V3	100	99.82	0.51	99.34	0.62	0.158
CASIA-V3	150	99.88	0.44	99.80	0.56	0.118
CASIA-V3	200	99.96	0.40	99.88	0.51	0.039
CASIA-V3	250	100	0.37	99.88	0.51	0.039
CASIA-V1	50	96.29	0.73	87.03	0.81	0.855
CASIA-V1	100	98.14	0.63	96.29	0.72	0.069
CASIA-V1	150	100	0.56	98.14	0.66	0.035
CASIA-V1	200	100	0.52	99.07	0.62	0.021
CASIA-V1	250	100	0.49	99.07	0.59	0.011
UBATH	50	100	0.60	100	0.72	0.000
UBATH	100	100	0.52	100	0.56	0.000
UBATH	150	100	0.44	100	0.47	0.000
UBATH	200	100	0.39	100	0.43	0.000
UBATH	250	100	0.35	100	0.39	0.000
IITD	50	93.44	0.80	90.30	0.85	1.709
IITD	100	97.81	0.70	95.01	0.75	0.672
IITD	150	98.93	0.63	97.36	0.69	0.448
IITD	200	98.93	0.57	98.03	0.65	0.444
IITD	250	99.38	0.54	98.26	0.61	0.260

**Table 5:** Accuracy analysis on the CASIA-V3 dataset at 16,384-dimensional (64×256) features, with PCA trained on 50% of subjects.

Database	PCA Dimensions	TAR at 0.1% FAR	Threshold at 0.1% FAR	TAR at 0.01% FAR	Threshold at 0.01% FAR	EER%
CASIA-V3	50	95.19	0.73	99.42	0.63	0.192
CASIA-V3	100	100	0.51	99.80	0.60	0.024
CASIA-V3	150	100	0.45	99.80	0.54	0.005
CASIA-V3	200	100	0.40	100	0.499	0.000
CASIA-V3	250	100	0.37	100	0.46	0.000

## Data Availability

This study utilized a publicly available dataset.

## Abbreviations

The following abbreviations are used in this manuscript:

FHE	Fully Homomorphic Encryption
PCA	Principal Component Analysis
EER	Equal Error Rate
NN	Nearest neighbor
MCP	Multi-class perceptron
PNN	Private nearest neighbor
PMCP	Private multi-class perception
IP	Inner product
HD	Hamming distance
MA	Matching algorithm
TAR	True Acceptance Rate
FAR	False Accept Rate
IITD	Indian Institute of Technology, Delhi
CASIA	Institute of Automation, Chinese Academy of Sciences
ISO	International Organization for Standardization
